# Bioinformatic Analysis Reveals an Immune/Inflammatory-Related Risk Signature for Oral Cavity Squamous Cell Carcinoma

**DOI:** 10.1155/2019/3865279

**Published:** 2019-12-13

**Authors:** Shuang Bai, Ying-Bin Yan, Wei Chen, Ping Zhang, Tong-Mei Zhang, Yuan-Yuan Tian, Hao Liu

**Affiliations:** ^1^Department of Oral and Maxillofacial Surgery, Stomatological Hospital of Nankai University, Tianjin Stomatological Hospital, No. 75, Dagu Road, Heping District, Tianjin 300041, China; ^2^Department of Oral and Maxillofacial Surgery, Peking University School and Hospital of Stomatology, No. 22, Zhongguancun South Avenue, Haidian District, Beijing 100081, China

## Abstract

High-throughput gene expression profiling has recently emerged as a promising technique that provides insight into cancer subtype classification and improved prediction of prognoses. Immune/inflammatory-related mRNAs may potentially enrich genes to allow researchers to better illustrate cancer microenvironments. Oral cavity squamous cell carcinoma (OC-SCC) exhibits high morbidity and poor prognosis compared to that of other types of head and neck squamous cell carcinoma (HNSCC), and these differences may be partially due to differences within the tumor microenvironments. Based on this, we designed an immune-related signature to improve the prognostic prediction of OC-SCC. A cohort of 314 OC-SCC samples possessing whole genome expression data that were sourced from The Cancer Genome Atlas (TCGA) database was included for discovery. The GSE41613 database was used for validation. A risk score was established using immune/inflammatory signatures acquired from the training dataset. Principal components analysis, GO analysis, and gene set enrichment analysis were used to explore the bioinformatic implications. When grouped by the dichotomized risk score based on the signature, this classifier could successfully discriminate patients with distinct prognoses within the training and validation cohorts (*P* < 0.05 in both cohorts) and within different clinicopathological subgroups. Similar somatic mutation patterns were observed between high and low risk score groups, and different copy number variation patterns were also identified. Further bioinformatic analyses suggested that the lower risk score group was significantly correlated with immune/inflammatory-related biological processes, while the higher risk score group was highly associated with cell cycle-related processes. The analysis indicated that the risk score was a robust predictor of patient survival, and its functional annotation was well established. Therefore, this bioinformatic-based immune-related signature suggested that the microenvironment of OC-SCC could distinguish among patients with different underlying biological processes and clinical outcomes, and the use of this signature may shed light on future OC-SCC classification and therapeutic design.

## 1. Introduction

Oral cavity squamous cell carcinoma (OC-SCC) is the most common malignancy of the head and neck region (excluding nonmelanoma skin cancer). There has also been a recent dramatic rise in the incidence of oropharyngeal squamous cell carcinoma (OP-SCC) [1]. Anatomically, the mouth and oropharynx are separate areas that are adjacent to each other but do not overlap. There are differences in the associated mortality, etiology, risk factors, and even the biomarkers of squamous cell carcinomas located within these two major sites.

Many risk factors can contribute to OC-SCC. Tobacco is classified as a group 1 carcinogen for OC-SCC. Currently, tobacco smokers exhibit a 3.43 fold relative risk for OC-SCC compared to that of nonsmokers [2], and they possess a fully adjusted HR of 1.7 [3]. Alcohol has been established as an independent risk factor, and studies of nonsmokers have demonstrated a strong correlation and dose-response relationship between alcohol consumption and OC-SCC [4]. Additionally, the combined effect of smoking and alcohol consumption was greater if the relationship was multiplicative [5]. HPV, as a major etiological factor, contributes disproportionally to the formation and prognosis of squamous cell carcinoma formation at different sites within the head and neck region [6]. Specifically, the high-risk genotype HPV-16 accounts for the vast majority (about 90% to 95%) of HPV-positive OP-SCC, while the HPV type is more variable in OC-SCC [7, 8]. Additionally, a far more favorable outcome exists for HPV positive compared to that for HPV-negative OP-SCC [9].

The above three major risk factors may contribute to carcinogenesis through different biological processes or pathways [10–12]; however, the dysregulation of immune or inflammatory responses is mutually shared among the carcinogenic mechanisms. Moderate alcohol consumption or alcohol abuse can suppress multiple arms of the immune response [13, 14]. Cigarette smoke exposure significantly affects the immune system, impairing the ability of the host to produce appropriate immune and inflammatory responses, ultimately leading to smoking-related pathologies [15]. These concepts are consistent with the notion that head and neck cancer is an intrinsically immune-suppressing disease [16]. For HPV-positive SCC, more intense PD-L1+, CD4+, and CD8+ T-cell infiltration correlates with a better outcome [17]. Increasing evidence supports the idea that evoked immune or inflammatory responses may elicit antitumor effects concerning OC-SCC development [18, 19].

Our current study aimed to develop a key gene signature that is representative of immune and inflammatory responses that could be correlated with patient prognosis, and we incorporated a bioinformatics-based approach associated with clinical covariates to achieve our aim. Following this principle, we performed a combined analysis to identify a robust gene signature, and we established a risk score system. Further bioinformatic analyses revealed that the risk score exhibited excellent prognostic value for stratifying patients irrespective of mutation or copy number variation (CNV) patterns, and this risk score was highly associated with cell-cycle processes.

## 2. Materials and Methods

### 2.1. Datasets

Whole genome mRNA expression RNA-seq (20101 genes) data, somatic mutation data, copy number variation data, and all corresponding clinical information from the TCGA-HNSC dataset [20] (http://cancergenome.nih.gov/) were downloaded for use as the training cohort. The following dataset was obtained for validation: GSE41613 (23520 genes) [21] (http://www.ncbi.nlm.nih.gov/geo/query/acc.cgi?acc=GSE41613). The training dataset comprised 314 OC-SCC patients while the validation dataset comprised 97 OC-SCC patients. The patient characteristics are summarised in Table S1. Two gene sets (immune response, M14329 and inflammatory response, M13657) [22] were extracted from the Molecular Signatures Database v6.1 (http://http://software.broadinstitute.org/gsea/msigdb/index.jsp) and were combined to integrate the immune/inflammatory-related gene set.

### 2.2. Statistical Analysis

Overall survival time (OS) was defined as the interval from the date of diagnosis until death or until the last follow-up. The prognostic value for patients possessing high or low expression of a certain gene or score (higher or lower than the median value) was calculated using the Kaplan–Meier method with the two-sided log-rank test by package “survival” of R. Univariate and multivariate COX regression analysis was also performed using package “survival” of R. Chi-squared test and Fisher's exact test were used to compare the frequencies between groups. A two-tailed Student's *t*-test was performed to compare two groups of numerical values. Analysis of variance (ANOVA) was used to analyze the differences among group means. The median absolute deviation (MAD) calculated in R. Pearson correlation analysis was used to evaluate the association between two variables and was calculated by *R* function “cor.test.” The statistical analysis was performed using the software of *R* version 3.4 for Windows. The statistical significance was established at the level of *P* < 0.05.

### 2.3. Bioinformatic Analysis

The *R* “Limma” package, a package that can perform the differential expression analyses of RNA sequencing (RNA-seq) data [23], was used to identify differentially expressed genes (DEGs) based on a threshold of false discovery rate (FDR) of less than 0.05. The packages “gaia,” “maftool,” and “circlize” were used to generate mutation and CNV plots [24]. The *R* “TCGAbiolinks” package was employed to investigate relevant biological implications [25]. The biological phenotype was further verified by gene set enrichment analysis (GSEA) [22]. Normalized enrichment score (NES) and false discovery rate were used to determine the statistical significance. The *R* “ESTIMATE” package that was fitted for our data was used to calculate ImmuneScore, StromalScore, and tumor purity [26].

## 3. Results

### 3.1. Different Immune/Inflammatory Phenotypes of OC-SCC Tumor

**Figure 1:**
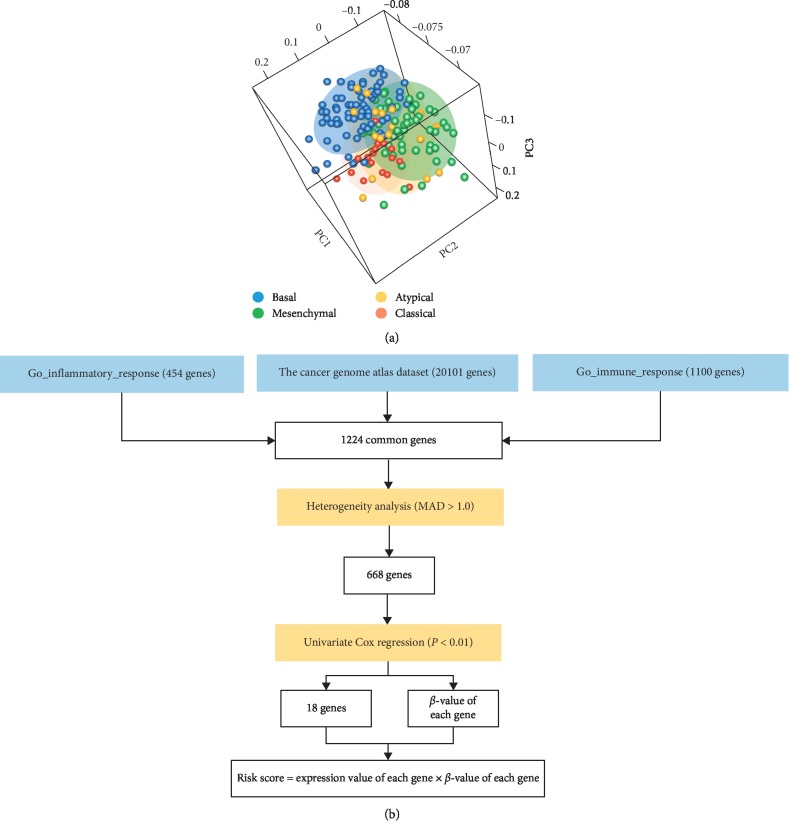
Different immune/inflammatory patterns of OC-SCC subtypes and the workflow of the signature establishment. (a) Principal components analysis of immune/inflammatory genes among four subtypes of OC-SCC. (b) Workflow of the signature establishment.

The gene expression and clinical data for 314 patients were obtained from the TCGA database (Table S1). Previous research defined four RNA subtypes of the TCGA cohort, including atypical, basal, classical, and mesenchymal [20]. The combined gene set that was representative of immune/inflammatory response (1224 genes) was used to illustrate the immune microenvironment of the OC-SCC tumor. Principal components analysis based on the 1224 genes revealed a different distribution pattern regarding OC-SCC tumor subtypes. A mutually exclusive pattern was observed within the mesenchymal, basal, and classical subtypes, while the atypical subtype lacked a clear distribution pattern (Figure 1(a)). As clinical differences between the four tumor subtypes were previously established, we sought to construct a gene signature to further explore the immune phenotype of OC-SCC tumors.

### 3.2. Identification of an Immune/Inflammatory Signature for Prognosis Prediction in OC-SCC

**Figure 2:**
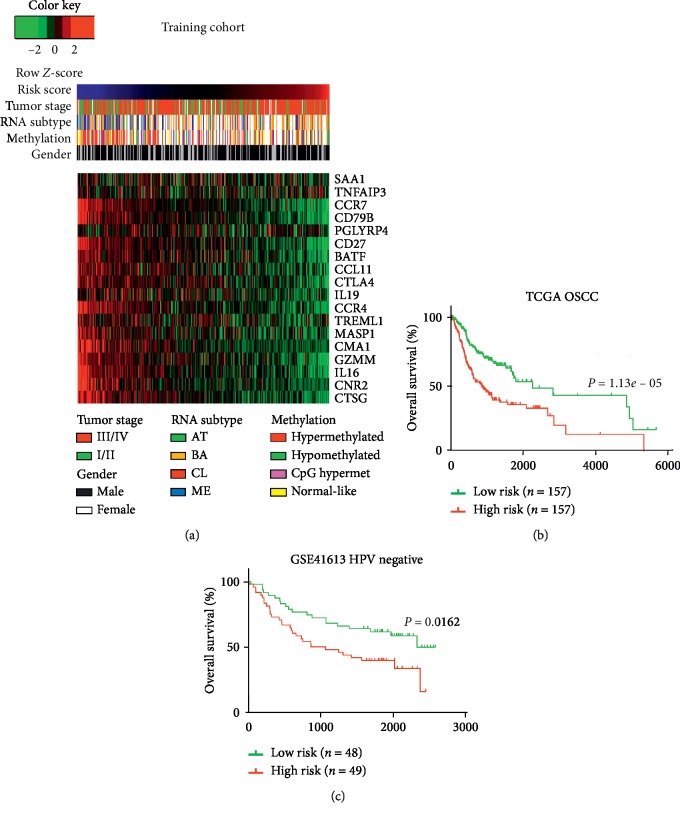
Overview of the eighteen-gene-based risk score and its prognostic value across different cohorts. (a) Heatmap depicting the gene expression values of the eighteen genes comprising the signature of the training cohort. Columns representing each sample that were sorted by increasing value of the risk score. Rows representing the expression value of each gene. (b) Kaplan–Meier survival analyses based on the median cutoff of the risk score within the training dataset. (c) Kaplan–Meier survival analyses based on the median cutoff of the risk scores within the validation dataset.

Taking into account the differential distribution of immune/inflammatory genes among OC-SCC subtypes, we sought to identify the gene or group of genes that were of significant prognostic value. As the PCA exhibited a heterogeneous expression pattern among OC-SCC patients, genes that were homogeneously expressed (MAD ≤ 1) were excluded from further analysis. Univariate Cox regression analysis was used to explore the prognostic value of the resulting genes. Eighteen genes (CD27, CD79B, CMA1, CCR4, CCR7, CNR2, CTLA4, CTSG, GZMM, IL16, MASP1, SAA1, CCL11, TNFAIP3, BATF, IL19, PGLYRP4, and TREML1) were identified to be associated with prognosis in OC-SCC patients (Figures 1(b) and 2(a)).

Next, the risk score method was employed based on gene expression levels (coefficient of each gene was the beta value in the univariate Cox regression model of each gene; Table S2), and the associations between TCGA results and two functional gene sets were explored. A strong correlation was identified between the risk score and the ssGSEA score in the immune/inflammatory response gene set (*R* = −0.693, *P* < 0.0001; Figure S1A). The resulting 18 genes were also mutually correlated, and this supported the integrity of the signature genes (Figure S1B and S1C). Patients were divided into high-risk and low-risk groups based on the median cutoff value of the risk score. This classifier could stratify patients according to distinct prognosis within the training cohort (median OS = 804 vs 2166 days; *P*=1.13*e* − 05; Figure 2(b)) and within the validation cohort (*P*=0.0162; Figure 2(c)).

### 3.3. Distribution and Prognostic Value of the Risk Score among Subgroups of OC-SCC

The patients within the training cohort were further stratified based on several clinicopathological factors, including age, gender, stage, RNA subtype, and methylation subtype. We found that in patients classified at a higher stage, the classical and hypomethylated subtypes exhibited a higher risk score (Figure S2A). Based on the median cutoff value of risk score in the training cohort, the patients were divided into either a high- or low-risk group within each subgroup to query the prognostic value. A nearly universal result was achieved among most of the subgroups (Figure S2(B–J)), demonstrating that an elevated risk score was strongly correlated with poor prognosis and vice versa. Similar results were observed for the validation GSE41613 cohort (Figure S3). Univariate and multivariate Cox regression analyses also indicated that the risk score provided an independent prognostic factor after adjusting for other clinical covariates (Table 1). Of note, tobacco use, alcohol consumption, and HPV infection, when assessed as initiating risk factors for OC-SCC, did not provide significant HR value in either univariate or multivariate Cox analyses. We further explored the relationship between tobacco use, alcohol consumption, or HPV infection and the risk score, and we found that there were no significant differences.

### 3.4. Mutation and CNV Patterns of Different Subgroups of the Risk Score

To further investigate the impact of the risk score at the DNA level, TCGA cases possessing available somatic mutation and copy number variation (CNV) information were analyzed. Based on the increasing risk score, cases were, respectively, divided into four subgroups, and the most representative subgroups (lower quantile risk score group, *n* = 78 and higher quantile risk score group, *n* = 78) were selected.

**Figure 3:**
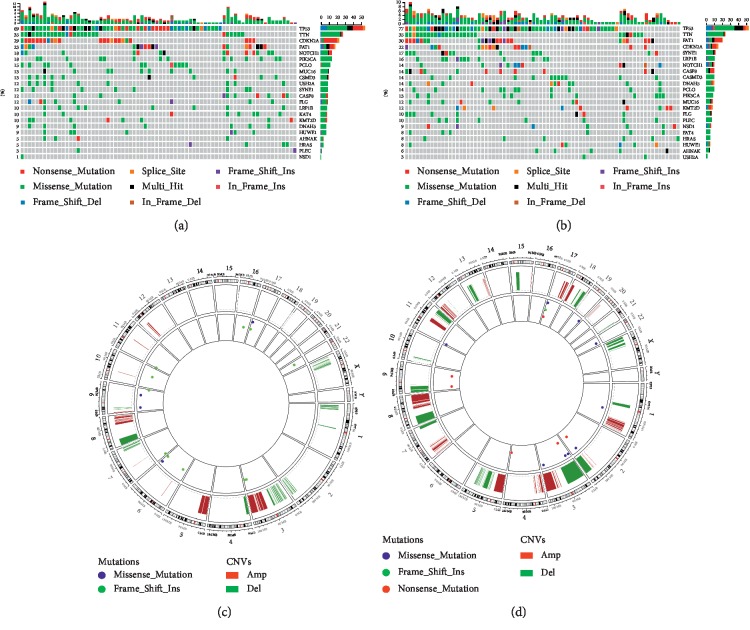
Different mutation and copy number variation patterns of the risk score. (a, b) An analysis of the 20 most mutated genes of either subgroup ((a), lower risk score; (b), higher risk score) was performed. Columns are sorted by samples with increasing risk score. (a) The sum of mutations in each of the sample categories is indicated by the legend; (b) the sum of the mutations in each gene is indicated by the legend. (c, d) The overall recurrent copy number variation (CNV) profile in order of increasing risk score ((c), lower risk score; (d), higher risk score).

An analysis of the 20 most frequently mutated genes within either subgroup was performed (Figure 3). Well-known mutated genes such as TP53, a somatic mutation detected in 60–80% of OSCC [27], were a genome guardian and played a pivotal role in regulating the cell cycle, cellular differentiation, DNA repair, and apoptosis and showed no significant mutation difference. There was also no significant difference regarding FAT1 mutation, which played a role in regulating the migration and invasion of OSCC cells through the localization of *β*-catenin [28]. Only frequent mutations in NSD1 (*P*=0.034) were significantly enriched in cases with higher risk score. Subsequently, CNV data were investigated, and our results revealed similar overall variant counts between OC-SCC at both lower and higher risk scores (mean 108.2 variants vs 111.6 variants; *P*=0.8713). There were some differences in the gene-level CNV landscape; however, the frequently deleted genomic regions were located at the 9p21.3 region encompassing the CDKN2A/CDKN2B (mean deletion −0.002 vs −0.138, *P*=0.015). The 7p11.2 region encompassing EGFR (mean amplification 0.083 vs 0.212, *P*=0.016) was frequently amplified for cases with higher risk scores (Figure 3).

### 3.5. High Risk Score OC-SCC Exhibited Cell-Cycle-Related Gene Function While Low Risk Score Exhibited Immune/Inflammatory Responses

**Figure 4:**
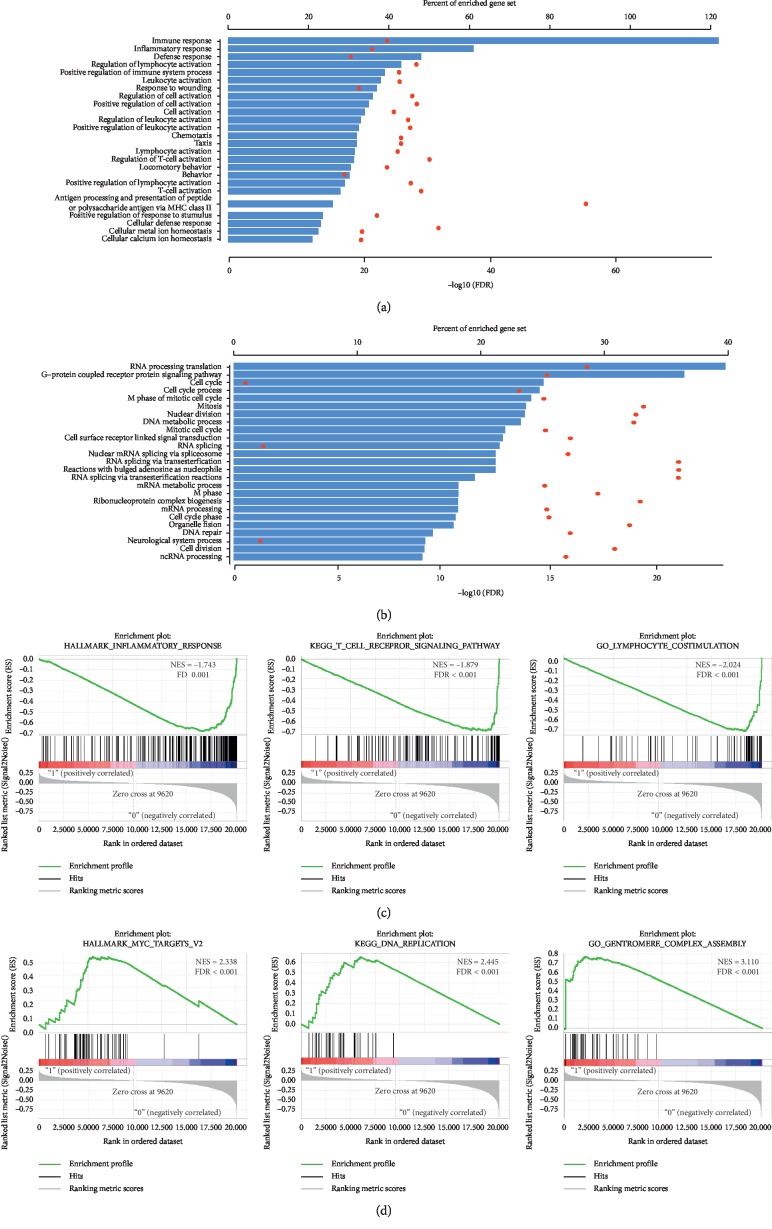
Biological annotation of the risk score. (a) GO results based on 1355 negatively correlated (*R* < −0.4) genes. (b) GO results based on 1632 positively correlated (*R* > 0.2) genes. (c) GSEA results of the lower risk score. (d) GSEA results of the higher risk score.

To further explore the prognostic value, similar patterns of mutation, and the CNV of the risk score, GO analysis was performed to assess the functional aspects. Pearson correlation score (*R*) was calculated for each gene within the training cohort. GO results based on 1355 negatively correlated (*R* < −0.4) genes suggested that these genes were highly enriched in immune/inflammatory responses (Figure 4(a)). Additionally, GO results based on 1632 positively correlated (*R* > 0.2) genes suggested that these genes were highly enriched in cell-cycle-related processes (Figure 4(b)). We next performed gene set enrichment analysis for further validation. GSEA revealed that a lower risk score was associated with processes or pathways closely related to immune/inflammatory responses (Figure 4(c)), and a higher risk score was highly correlated with cell-cycle-related processes (Figure 4(d)).

### 3.6. Association between the Risk Score and Tumor Purity

**Figure 5:**
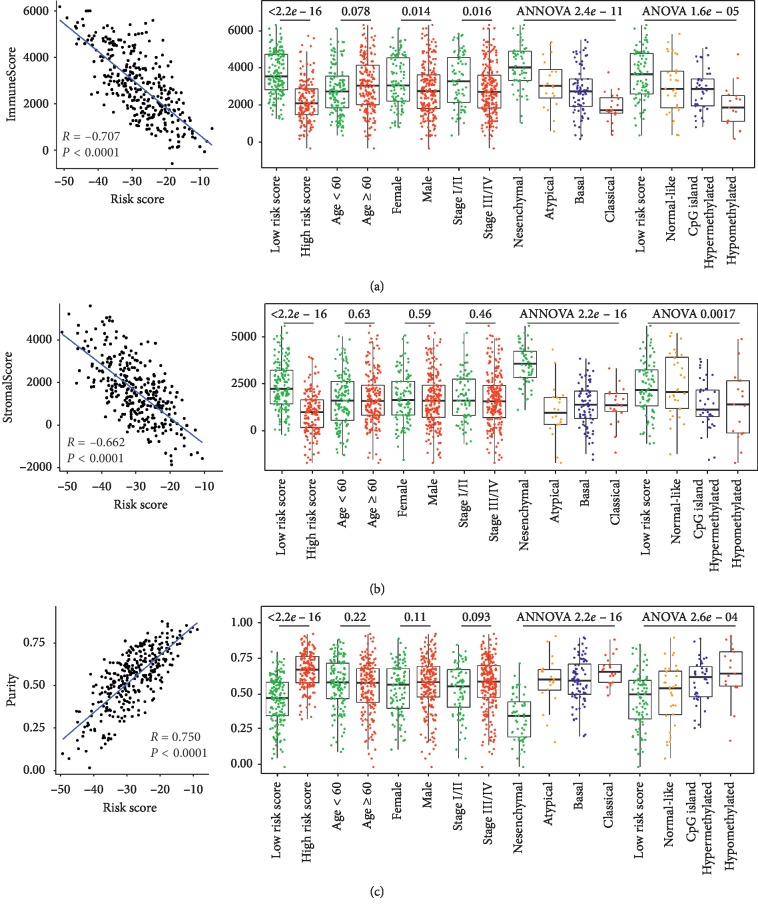
Association between the risk score and tumor purity. (a) Correlation between the ImmnueScore and the risk score and the distribution of the ImmnueScore among subgroups of OC-SCC within the training cohort. (b) Correlation between the StromalScore and the risk score and the distribution of the StromalScore among subgroups of OC-SCC within the training cohort. (c) Correlation between tumor purity and the risk score and the distribution of tumor purity among subgroups of OC-SCC in the training cohort.

It is established that OC-SCC tissues contain abundant nontumor cells within their microenvironment. Thus, tumor purity could reflect the tumor and nontumor compartments of the OC-SCC tissue. As analyzed above, the risk score was closely associated with immune/inflammatory responses. Based on this, we further explored the correlation between tumor purity and the risk score. The risk score exhibited a high negative correlation with ImmuneScore and StromalScore and a positive correlation with tumor purity (Figure 5). The results obtained using the validation cohort GSE41613 were in accordance with those obtained using the training cohort (Figure S4). These results further validated the idea that the risk score provided a robust predictor of OC-SCC immune/inflammatory responses; however, the dichotomized median value of either of the purity-related scores was unable to achieve a significant prognostic value.

## 4. Discussion

As the most common malignancy of the head and neck, OS-SCC is diagnosed using histopathological criteria and is staged using the TNM system [1]. Several studies have provided high-resolution images of the OC-SCC molecular landscape, and these images revealed significant changes that may contribute to the pathogenesis and biology of this disease [20]. Risk factors such as tobacco use, alcohol consumption, and HPV infection have been proposed as initiating risk factors for OC-SCC [29–31]; however, there is little consensus on how immune/inflammatory responses could affect OS-SCC subtypes. The development of meaningful signatures to determine the immune status of patients provides an attractive therapeutic approach to this disease, as these signatures not only promise to be powerful prognostic biomarkers, but if properly applied, they also stratify patients to increase the likelihood of positive outcomes in response to immunotherapy. In our study, we demonstrated that the four subtypes of OC-SCC represent distinct immune/inflammatory phenotypes, and based on this, we established a gene signature that could stratify patients to provide different prognoses.

For our signature, we investigated the DNA level scenario and discovered similar mutational and CNV patterns that were present in either high or low risk score groups. This result of the mutated genes suggested that it was the inflammatory responses and microenvironment that mainly contribute to the different prognosis rather than well-known tumorigenesis progresses. Only frequent mutations in NSD1 were significantly enriched in cases with higher risk scores. It was reported that NSD1 was more mutated in laryngeal and pharyngeal squamous cell carcinoma (L/P-SCC) than in OC-SCC [32], and given the role of NSD1 as a chromatin modifier, these mutations could contribute to cancer formation through a combination of rare germline variants and somatic loss-of-heterozygosity (LOH) [33].

Aneuploidy, also known as somatic copy number alterations, is widespread in human cancers and has been proposed to drive tumorigenesis. Aneuploidy was previously demonstrated to correlate with tumor cell proliferation and reduced immune processes [33]. In our study, overall copy number variant counts in high or low risk score cases were not significantly different. This suggested that our signature could infer immune/inflammatory responses irrespective of aneuploidy difference; however, some key genes did exhibit different variation patterns. The deletion of CDKN2A, a tumor suppressor gene that functions in G1 cell cycle control, was associated with poor prognosis and low survival rate in OC-SCC [34], and EGFR amplification was shown to be associated with advanced clinical stage in OC-SCC patients [35]. The result of the aneuploidy difference regarding CDKN2A and EGFR was in accordance with the worse prognosis higher risk score represented.

We performed GO and GSEA analyses to further validate the functional annotation of our signature. The results of these analyses indicated that a lower risk score was correlated with immune/inflammatory responses, and a higher risk score was associated with cell-cycle-related processes. These results could partially explain that patients possessing a higher risk score demonstrated a worse prognosis, and patients with more active immune/inflammatory responses exhibited a better outcome. The high correlation between the risk score and the tumor purity also suggested the presence of more immune cell infiltration in lower risk score cases. We also performed ssGSEA analysis to evaluate the relationship between our risk score and the tumor purity as defined by expression data. ImmuneScore, StromalScore, ESTIMATE score, and tumor purity were all significantly correlated with the risk score, and of these scores, the ImmuneScore exhibited the highest correlation. Of note, different from other cancer types such as glioma [36] and colon cancer [37], the purity-related scores or purity alone were unable to distinguish among different prognostic patient groups [38].

## 5. Conclusion

Taken together, we identified and validated an eighteen-gene-based immune/inflammatory signature that exhibited independent prognostic significance for patients with OC-SCC and reflected the overall intensity of immune/inflammatory responses within the tumor microenvironment. Our study offers new insights regarding the OC-SCC immune microenvironment and immune-related therapy for this disease. Evaluating this signature may help to elucidate the complex role of tumor immune/inflammatory responses in this disease and will provide new insight into clinical management and drug design.

## Figures and Tables

**Table 1:** COX regression analysis of the risk score and other characteristics in the TCGA OC-SCC cohort.

Variables	Univariate Cox regression		Multivariate Cox regression	
	HR	*P*	HR	*P*
Risk score (high vs low)	2.09	1.7*e* −* *05	1.99	0.0002
Age (>60 vs ≤60)	1.15	0.3963	1.31	0.1580
Gender (female vs male)	1.08	0.6698	1.10	0.6357
Tobacco history (yes vs no)	1.29	0.2099	1.27	0.2740
Alcohol history (yes vs no)	1.07	0.6958	1.01	0.9507
HPV status (positive vs negative)	0.88	0.7502	0.86	0.7138
Stage (III/IV vs I/II)	2.23	0.0006	2.10	0.0023

HR: hazard ratio.

## Data Availability

The preprocessed normalized expression data, CNV data, somatic mutation data, and clinical data of 314 OC-SCC (training cohort) samples belong to the TCGA dataset, retrieved from the website http://cancergenome.nih.gov/. The normalized expression data and clinical data of the GSE41613 dataset that comprised 97 OC-SCC samples were retrieved from the Gene Expression Omnibus website http://www.ncbi.nlm.nih.gov/geo/query/acc.cgi?acc=GSE41613.

## Supplementary Materials

Supplementary Materials Figure S1: association between the risk score and the immune genes and association among genes in the signature. Figure S2: distribution and prognostic value of the risk score across different subgroups of the training cohort. Figure S3: distribution and prognostic value of the risk score across different subgroups of the validation cohort. Figure S4: association between the risk score and tumor purity in the validation cohort. Table S1: demographic characteristics in the training cohort and validation cohort. Table S2: risk score formula.
