# Supplementary material for: Bioinformatic Analysis Reveals an Immune/Inflammatory-Related Risk Signature for Oral Cavity Squamous Cell Carcinoma
Source: J Oncol. 2019 Dec 13;2019:3865279. doi: 10.1155/2019/3865279 (PMC6930791; doi:10.1155/2019/3865279)
Supplement: Supplementary Materials — Figure S1: association between the risk score and the immune genes and association among genes in the signature. Figure S2: distribution and prognostic value of the risk score across different subgroups of the training cohort. Figure S3: distribution and prognostic value of the risk score across different subgroups of the validation cohort. Figure S4: association between the risk score and tumor purity in the validation cohort. Table S1: demographic characteristics in the training cohort and validation cohort. Table S2: risk score formula. [file 3865279.f1.pdf]

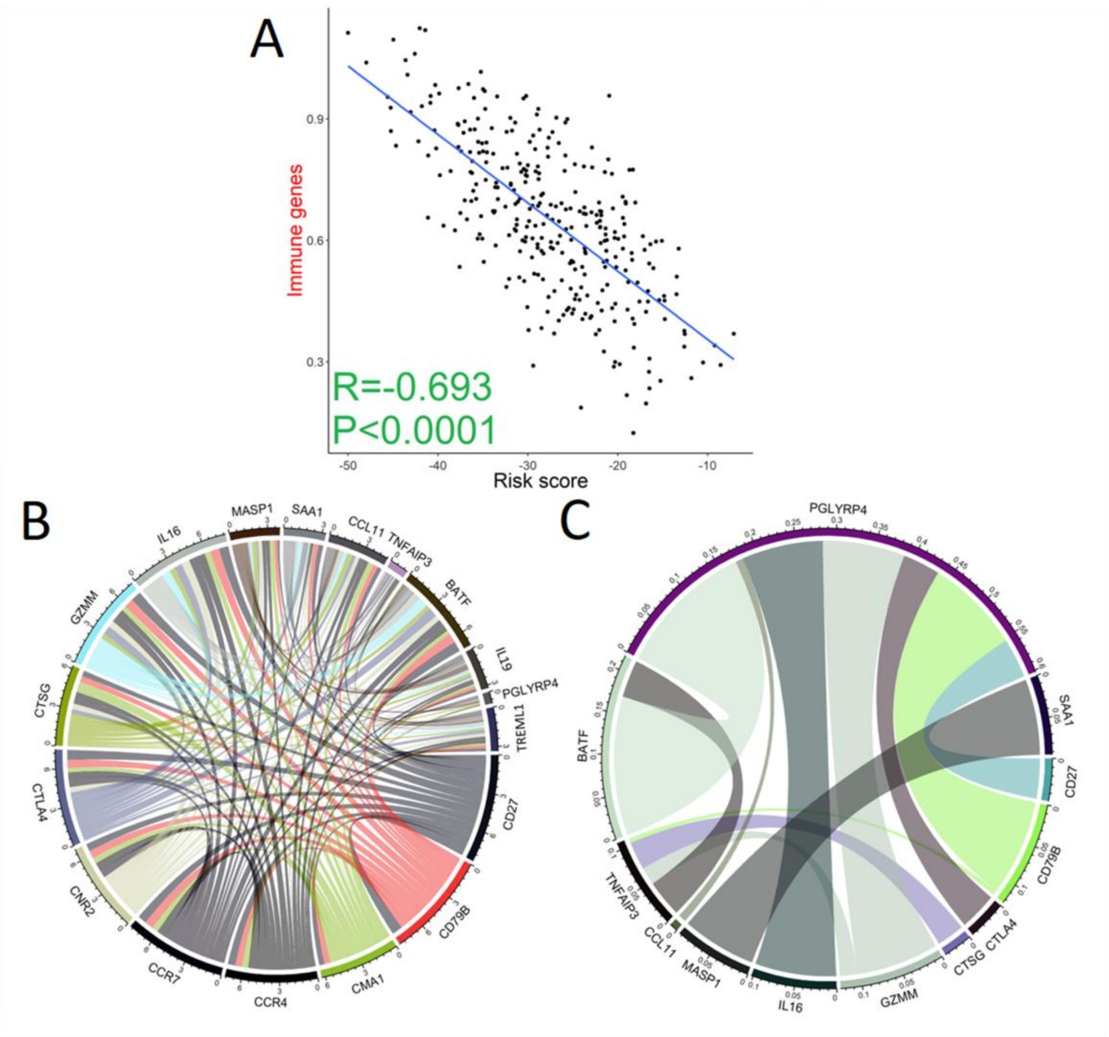

Fig. S1 Association between the risk score and the immune genes and association among genes in the signature.

(A) Association between the risk score and the immune genes in the training cohort. **(B)** Positive correlation among genes in the signature. Stripe width indicated the r value. **(C)** Negative correlation among genes in the signature. Stripe width indicated the r value.

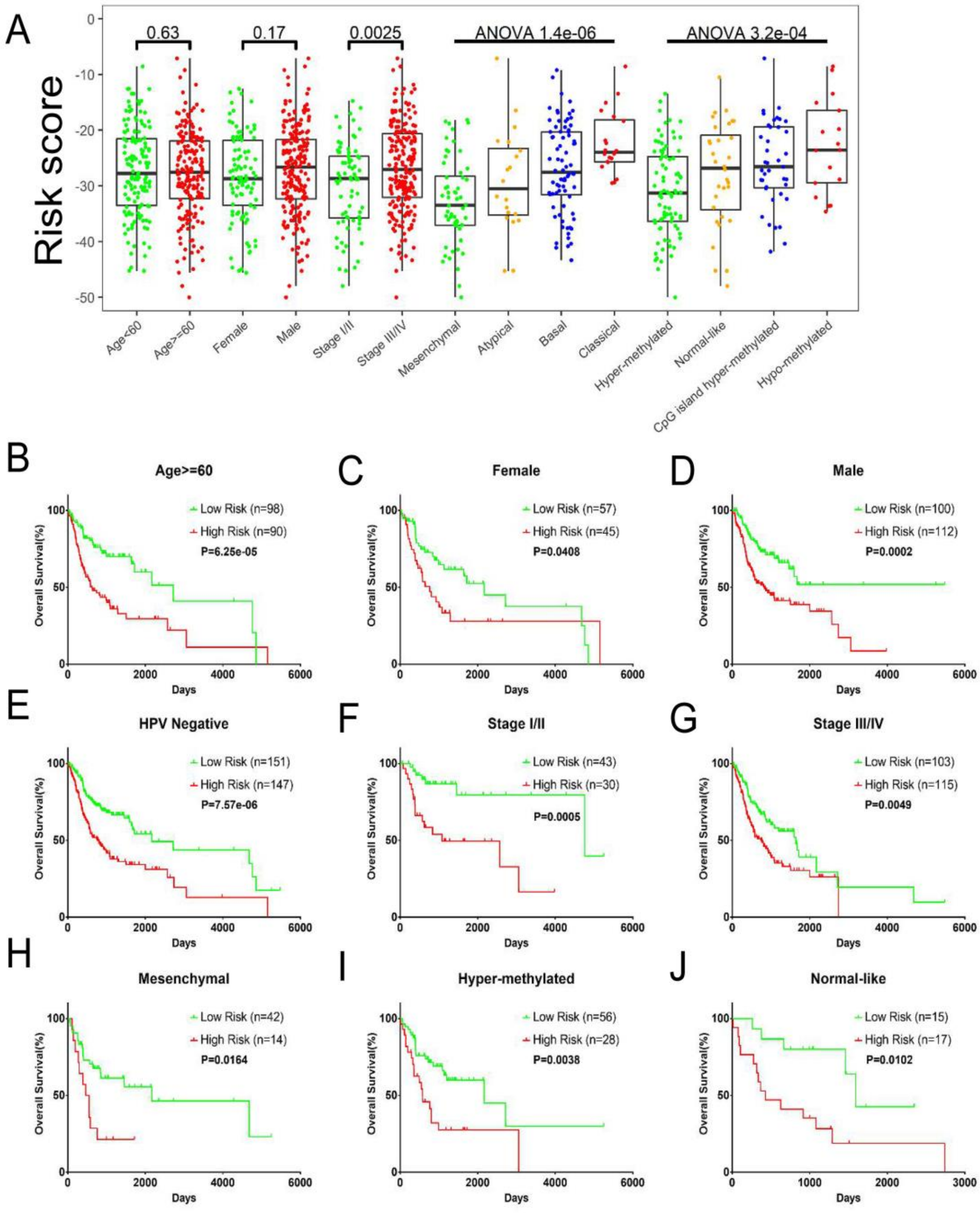

Fig. S2 Distribution and prognostic value of the risk score across different subgroups of the training cohort.

(A) Distribution of the risk score among subgroups of OC-SCC in the training cohort. **(B-J)** Kaplan–Meier survival analyses based on the dichotomized risk score defined by clinicopathologic factors of the training cohort.

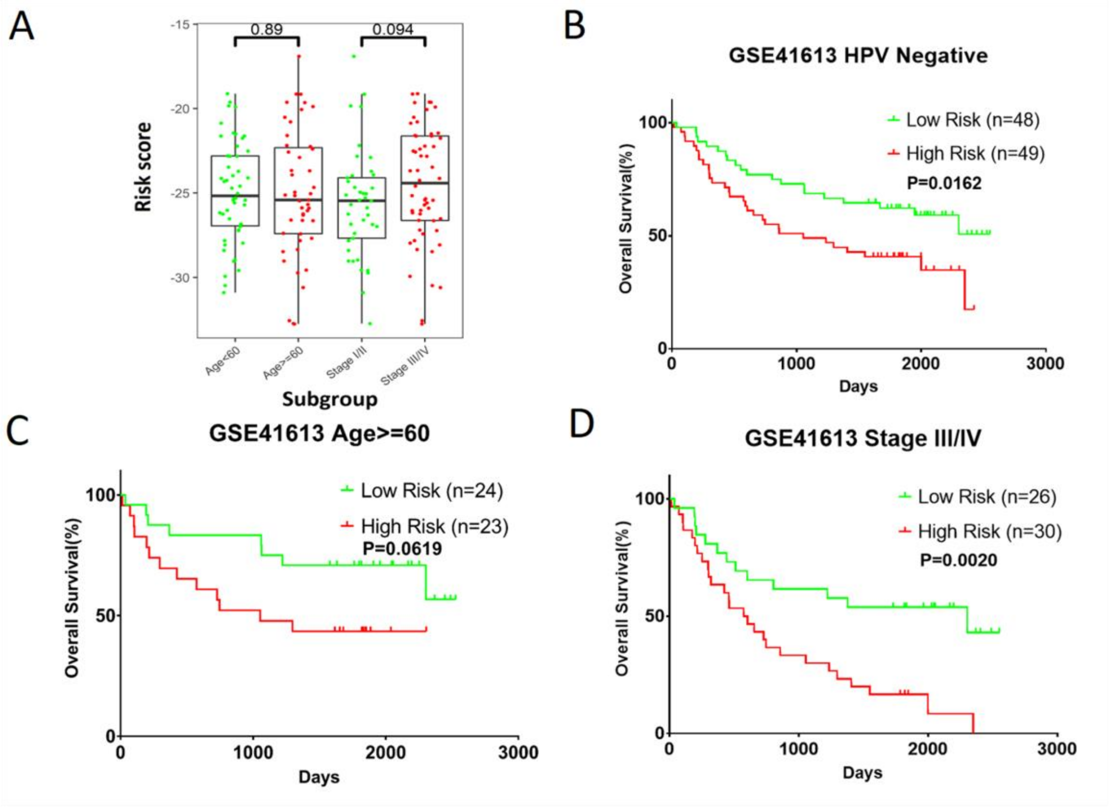

Fig. S3 Distribution and prognostic value of the risk score across different subgroups of the validation cohort.

(A) Distribution of the risk score among subgroups of OC-SCC in the validation cohort. **(B-D)** Kaplan–Meier survival analyses based on the dichotomized risk score defined by clinicopathologic factors of the validation cohort.

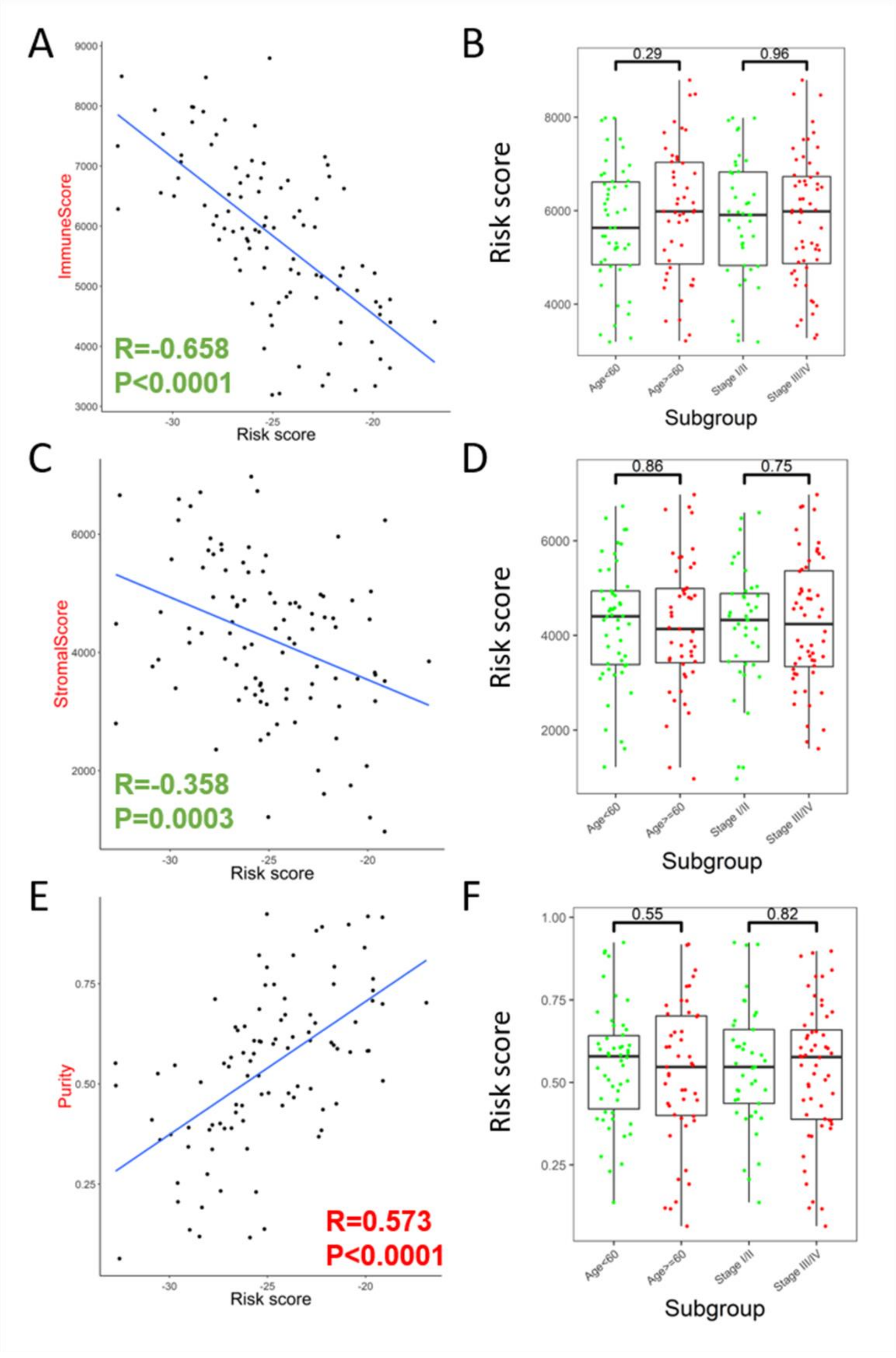

Fig. S4 Association between the risk score and tumor purity in the validation cohort.

(A, B) Correlation of ImmnueScore and the risk score and the distribution of the ImmnueScore among subgroups of OC-SCC in the validation cohort. **(C, D)** Correlation of StromaScore and the risk score and the distribution of the StromaScore among subgroups of OC-SCC in the validation cohort. **(E, F)** Correlation of tumor purity and the risk score and the distribution of tumor purity among subgroups of OC-SCC in the validation cohort.

Table S1. Demographic characteristics in the training cohort and validation cohort.

Patient characteristics	Training cohort (n=314)	Validation cohort (n=97)	*P* value
Age (year)			0.1911
<60	136	50	
≥60	178	47	
Gender			1.0000
Male	212	66	
Female	102	31	
Stage			0.0020
I/II	73	41	
III/IV	218	56	
Disease site			
OSCC	314	97	
Alveolar Ridge	18		
Buccal Mucosa	22		
Floor of mouth	62		
Hard Palate	7		
Lip	3		
Oral Cavity	73		
Oral Tongue	129		
HPV status			0.0877
HPV16 positive	12	0	
HPV33 positive	4	0	
HPV negative	298	97	
RNA subtype			
Atypical	20		
Basal	76		
Classical	20		
Mesenchymal	56		
Methylation subtype			
CpG island hyper-methylated	39		
Hyper-methylated	84		
Hypo-methylated	17		
Normal-like	32		

P value was calculated by Pearson chi-squared test or Fisher exact test depending on patient counts in each subgroup.

Table S2. Risk score formula.

Gene	Univariate Cox Regression	
	Beta	*P* Value
CD27	-0.444	0.009
CD79B	-0.439	0.009
CMA1	-0.510	0.003
CCR4	-0.473	0.005
CCR7	-0.437	0.009
CNR2	-0.557	0.001
CTLA4	-0.467	0.006
CTSG	-0.675	<0.001
GZMM	-0.518	0.002
IL16	-0.519	0.002
MASP1	-0.498	0.004
SAA1	0.475	0.005
CCL11	-0.466	0.006
TNFAIP3	0.442	0.009
BATF	-0.451	0.008
IL19	-0.470	0.006
PGLYRP4	-0.439	0.009
TREML1	-0.494	0.004

Risk score = (-0.444 × CD27 expression) + (-0.439 × CD79B expression) + (-0.510 × CMA expression) + (-0.473 × CCR4 expression) + (-0.437 × CCR7 expression) + (-0.557 × CNR2 expression) + (-0.467 × CTLA4 expression) + (-0.675 × CTSG expression) + (-0.518 × GZMM expression) + (-0.519 × IL16 expression) + (-0.498 × MASP expression) + (0.475 × SAA1 expression) + (-0.466 × CCL11 expression) + (0.442 × TNFAIP3 expression) + (-0.451 × BATF expression) + (-0.470 × IL19 expression) + (-0.439 × PGLYRP4 expression) + (-0.494 × TREML1 expression)
